# Identification of novel cyanoacrylate monomers for use in nanoparticle drug delivery systems prepared by miniemulsion polymerisation – A multistep screening approach

**DOI:** 10.1016/j.ijpx.2022.100124

**Published:** 2022-07-20

**Authors:** Astrid Hyldbakk, Yrr Mørch, Sofie Snipstad, Andreas K.O. Åslund, Geir Klinkenberg, Vu To Nakstad, Ane-Marit Wågbø, Ruth Schmid, Peter P. Molesworth

**Affiliations:** a Department of Biotechnology and Nanomedicine, SINTEF Industry, Trondheim, Norway; b Department of Physics, Norwegian University of Science and Technology, Trondheim, Norway; c Cancer Clinic, St. Olavs Hospital, Trondheim, Norway

**Keywords:** Poly(alkyl cyanoacrylate) nanoparticles, Drug delivery, Toxicity screening, Cabazitaxel, Nanoparticle characterisation

## Abstract

Poly (alkyl cyanoacrylate) (PACA) polymeric nanoparticles (NPs) are promising drug carriers in drug delivery. However, the selection of commercially available alkyl cyanoacrylate (ACA) monomers is limited, because most monomers were designed for use in medical and industrial glues and later repurposed for drug encapsulation. This study therefore aimed to seek out novel ACA materials for use in NP systems using a toxicity led screening approach. A multistep strategy, including cytotoxicity screening of alcohols as degradation products of PACA (44 alcohols), NPs (14 polymers), and a final *in vivo* study (2 polymers) gave poly (2-ethylhexyl cyanoacrylate) PEHCA as a promising novel PACA candidate. For the first time, this work presents cytotoxicity data on several novel ACAs, PEHCA *in vivo* toxicity data, and miniemulsion polymerisation-based encapsulation of the cabazitaxel and NR688 in novel PACA candidates. Furthermore, several of the ACA candidates were compatible with a wider selection of lipophilic active pharmaceutical ingredients (APIs) *versus* commercially available controls. Combined, this work demonstrates the potential benefits of expanding the array of available ACA materials in drug delivery. Novel ACAs have the potential to encapsulate a wider range of APIs in miniemulsion polymerisation processes and may also broaden PACA applicability in other fields.

## Highlights

• Screening of novel poly(alkylcyanoacrylate) (PACA) materials to broaden PACA nanomedicine potential.
• A comprehensive screening process evaluated the toxicity of novel poly(alkylcyanoacrylate) (PACA) materials.
• Novel poly(2-ethylhexyl cyanoacrylate) nanoparticles has a promising safety profile.
• Novel ACA materials show potential to enable encapsulation of a wider range of APIs.

## Graphical abstract

**Figure:**
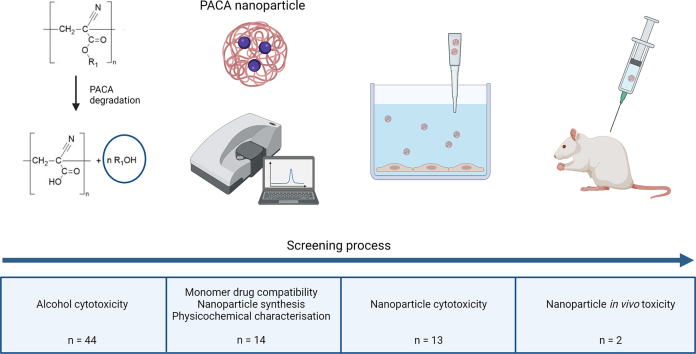

## 1 Introduction

Poly (alkyl cyanoacrylate) (PACA) based materials play a key role in the nanomedicine field and have been used in multiple late-stage clinical trials (Vauthier, 2019; Merle et al., 2019; Kumari et al., 2010; Bayer, 2018). The interest in polymeric nanoparticles (NPs) began in 1979, when the young pharmacist Patrick Couvreur borrowed surgical glue from his surgeon colleagues and formed PACA NPs as the first biodegradable polymeric NPs ever produced (Couvreur et al., 1979a). He was intrigued by this material's biocompatible and biodegradable properties, high ability of self-polymerisation, well-defined degradation properties, and he showed that PACA NPs were able to encapsulate poorly water-soluble drugs with high loading capacities (Couvreur et al., 1979a; Couvreur et al., 1979b). Couvreur used polymethyl- and polyethyl-cyanoacrylates, using sorption to load the active pharmaceutical ingredients (APIs) onto the preformed nanoparticles.

Subsequently, ACA monomers have been used for encapsulation and delivery of a wide range of APIs, including small molecules, proteins, RNA and DNA. Preparation of PACA NPs has been achieved either directly using emulsion/miniemulsion, interfacial, micellar polymerisation processes or by pre-polymerisation of the monomer followed by NP formation through self-assembly and precipitation processes (Vauthier, 2019). APIs can be introduced using a wide variety of methods ranging from solubilisation or suspension in the monomer prior to emulsification, solubilisation in either phase during self-assembly/precipitation methods or *via* sorption or attachment to pre-formed NPs. This flexibility is in partly why PACA systems have proved so popular (Vauthier, 2019; Vauthier et al., 2007; Arpicco et al., 2016; Krauel et al., 2004).

Miniemulsion polymerisation is one of the more widely used encapsulation processes for preparing PACA NPs and has been a focus in our earlier work with hydrophobic API (Fusser et al., 2019; Snipstad et al., 2017; Sulheim et al., 2017). During miniemulsion polymerisation, it is crucial to ensure good compatibility between the ACA and the API to allow for a controlled and reproducible NP synthesis and for the API to be encapsulated without covalent bonding to the polymer (with concurrent reduction in encapsulation efficiency or formation of a prodrug). ACA monomers are typically very reactive, and polymerise rapidly in the presence of drug compounds containing nucleophilic features such as anions (hydroxide, iodide, *etc.*) or weak bases (alcohol, amine, *etc.*) (Fig. S1-A) (Nicolas and Couvreur, 2009). Low reactivity between the ACA and the API is therefore important to avoid premature polymerisation. Additionally, whilst not essential for successful encapsulation, one of many considerations when designing a nanoparticle delivery system is that the grade of API solubility in the ACA has a great influence on the loading, encapsulation efficiency, degree of API protection, and release rate/release profile of the API (Krauel et al., 2004; Huang and Lee, 2006).

Throughout the last decades, the PACA family has expanded to include several monomers with different side chains. However, as alkyl cyanoacrylates (ACAs) are still mainly used as adhesives, the range of commercially available ACA monomers is limited and dominated by glue manufacturers. Therefore, most drug delivery research to date has focused on the monomers readily available on the open market, including, ethyl cyanoacrylate (ECA), n-butyl cyanoacrylate (BCA), 2-ethyl-butyl cyanoacrylate (2-EBCA), 4-methyl-pentyl cyanoacrylate/isohexyl cyanoacrylate (4-MPCA/IHCA)^1^ and n-octyl cyanoacrylate (OCA). The choice of ACA monomer used in the polymerisation process dictates many of the NP key properties, including polymerisation kinetics, particle morphology, degradation rate, drug compatibility and toxicity profile (Vauthier et al., 2007; Nicolas and Couvreur, 2009; Szwed et al., 2019; Müller et al., 1992; Lherm et al., 1992). Using the optimal ACA monomer for the specific application of interest is therefore crucial to get a successful result. Raised awareness regarding toxicity of drugs, additives and compounds used in medical delivery systems has led to an increased focus on approaches assessing nanotoxicity in biological systems and to include the safety aspect early in the development process, safety-by-design. Still, evaluating NP toxicity is challenging, as the toxic effects can originate from both intact particles and degradation products. Seemingly small changes in NP size, shape, surface charge, surface molecules and core materials can induce dramatic differences in potency and safety, making it hard to predict the outcome of NP interactions with biological systems. It is therefore crucial to characterise NP interactions in cellular assays and animal models. In this study we therefore aimed at identifying novel ACA monomers suitable for the preparation of PACA NP drug delivery systems by miniemulsion polymerisation, mainly based on polymer toxicity and drug compatibility.

We present the results from a multistep screening process, focusing on PACA NP toxicity and ACA drug compatibility, both in cell lines and in a rat animal model. Our starting point was the cellular toxicity of the NP degradation products. The biological degradation of PACA occurs mainly *via* hydrolysis of the ester side groups, leading to the release of an alcohol (Fig. S1-B). As the only chemical variations between PACA polymers lies in the side groups, the released alcohol is specific for each PACA. Additionally, the hydrolysis rate will differ between each PACA, as it is based upon the hydrophobicity of the ester side group, which in turn is affected by the number of carbons and substitution pattern of the side group. In a biological system, PACA NPs can degrade both intra- and extracellularly, and alcohols may therefore interact with and penetrate both cellular and subcellular (*e.g.* endosomal) membranes depending on their chain length and structure (Kondela et al., 2017; Wanderlingh et al., 2010; Patočka and Kuča, 2012). This can potentially lead to toxic effects by alcohol accumulation in specific tissues after prolonged treatments with PACA formulations. While accepting that degradation rates would not be taken into account with the proposed toxicity screening approach, it was hypothesised that it would be possible to rapidly exclude PACA materials that would form alcohols with high toxicity levels upon accumulation. As PACA NPs are shown to accumulate in liver tissue (Fusser et al., 2019; Snipstad et al., 2017), emphasis was put on alcohol toxicity in liver cells. We compared the cytotoxicity of 44 primary and secondary alcohols, using the alcohols corresponding to the commercially available alternatives BCA, 2-EBCA, 4-MPCA and OCA as controls. 2-EBCA results were prioritised as PEBCA NPs have been shown to have promising physicochemical properties and a low toxicity profile after thorough characterisation by our research group and our collaborators (Sulheim et al., 2017; Nicolas and Couvreur, 2009; Huang and Lee, 2006). Additionally, PEBCA NPs have reached phase III clinical trials for treatment of liver cancer (Merle et al., 2019).

From the alcohol screening results, 14 corresponding monomers were chosen for drug compatibility testing with 5 relevant drugs, and miniemulsion polymerisation-based NP synthesis both with and without loading of the model drugs cabazitaxel and NR668. The empty NP batches underwent a second cellular toxicity screening, from where the lead candidate poly (ethylhexyl cyanoacrylate) (PEHCA) was evaluated in a final *in vivo* toxicity study in rats. To our knowledge, this is the first time that 8 novel PACAs were tested for toxicity in cells, that they are shown to encapsulate cabazitaxel and NR668 and that PEHCA NPs are studied in an animal model. Combined, these screening steps formed a multistep strategy with potential as a cost-effective methodology to indicate the type of PACA materials that could be used in drug delivery applications.

PACA NPs are, based on their physicochemical characteristics, an interesting platform for sustained drug delivery in the peritoneal cavity. We have recently shown that local intraperitoneal (i.p.) administration of drug-loaded NPs can ensure a higher local drug concentration and decreased systemic toxicity compared to that of injection of free drug (Fleten et al., 2021). This is a new and potentially more efficient treatment regime for cancer types in the peritoneal cavity, such as peritoneal metastases (Morch et al., 2022). Based on these findings, the final *in vivo* toxicity study of PACA NPs was performed with i.p. injection as administration route.

## 2 Materials and methods

### 2.1 Cellular toxicity of alcohols

Alcohols were purchased from Sigma Aldrich (Sigma-Aldrich, an affiliate of Merck KGaA, Darmstadt, Germany), VWR International LLC (VWR International, LLC, Kokstad, Norway), at the highest purity reasonably available and were used as received.

The cell lines used for determining alcohol cytotoxicity were Hep G2 (ATCC HB-8065, human hepatocyte carcinoma cells) and LLC-PK1 (ATCC CL-101, porcine epithelial kidney cells). Both cell lines were obtained from American Type Culture Collection (ATCC) and were chosen as they are part of the standardised nanomaterial cytotoxicity profiling regime used by the Nanotechnology Characterization Laboratory (NCL) (Nanotechnology Characterization Laboratory (NCL), 2021a; Nanotechnology Characterization Laboratory (NCL), 2021b). Hep G2 was cultivated in RPMI 1640 (Gibco) supplemented with 10% (v/v) fetal bovine serum (FBS, Sigma). LLC-PK1 was cultivated in Medium 199 (Sigma) supplemented with 3% (v/v) FBS (Sigma). In addition, both media were supplemented with 2 mM l-Glutamine (Gibco) and 100 units/mL penicillin/streptomycin (Gibco). The cells were cultivated in 384 well plates (Corning 3712), were 30 μL/well corresponded to 6 × 10^3^ or 3.62 × 10^3^ seeded cells/well for Hep G2 and LLC-PK1, respectively. The cells were cultivated in humidified atmosphere at 37 °C and 5% CO_2_ overnight, before diluted alcohols were added the next day (5 μL/well) to yield final alcohol concentrations ranging from 0.01 to 3% (v/v) in cell culture medium. The alcohols were diluted in cell culture medium prior to addition to the cell cultures. Cells were incubated for 24 h, before the toxicity was assessed using the CellTiter-Glo® 2.0 Luminescent Cell Viability Assay (CTG 2.0, Promega). This assay uses ATP as a marker for viable cells and the amount of ATP is quantified using firefly luciferase. After ended incubation, 0.5× CTG 2.0 diluted in PBS was added to 36% (v/v) of final volume in all wells followed by mixing (1800 rpm, Bioshake) and thereafter 10 min incubation before the luminescent signal was recorded using a luminometer (SpectraMax I3X, Molecular Devices). Cell seeding and addition of diluted alcohols were performed using a Tecan Freedom EVO 200 robotic system.

### 2.2 ACA monomers

Monomers were purchased from Cuantum Medical Cosmetics (Spain) or as gift from Loctite (Ireland). An overview of the monomers is presented in Table 1 and Table S1 (includes chemical structures).

**Table 1:** Overview of ACA monomers used in this study, including their abbreviation, full name and corresponding alcohol. The monomers are listed in an alphabetical order.

ACA monomer		Corresponding alcohol degradation product
BCA	Butyl cyanoacrylate	1-butanol
3,3-DMBCA	3,3-dimethylbutyl cyanoacrylate	3,3-dimethyl-1-butanol
2-EBCA	2-ethylbutyl cyanoacrylate	2-ethyl-1-butanol
2-EHCA	2-ethylhexyl cyanoacrylate	2-ethyl-1-hexanol
1-HPCA	1-heptyl cyanoacrylate	1-heptanol
2-HPCA	2-heptyl cyanoacrylate	2-heptanol
3-HPCA	3-heptyl cyanoacrylate	3-heptanol
3-MBCA	3-methylbutyl cyanoacrylate	3-methyl-1-butanol
4-MPCA	4-methylpentyl cyanoacrylate	4-methyl-1-pentanol
NPCA	Neopentyl cyanoacrylate	Neopentanol
OCA	Octyl cyanoacrylate	1-octanol
1-PCA	1-pentyl cyanoacrylate	1-pentanol
3-PCA	3- pentyl cyanoacrylate	3-pentanol
2-PECA	2-phenylethyl cyanoacrylate	2-phenylethanol

### 2.3 Drug solubility

Five selected drugs were tested for their solubility in ACA monomers: docetaxel, mupirocin, cabazitaxel (CBZ), belinostat (Biochempartner Co. Ltd., China) and oxaliplatin (AKScientic, CA, USA). The solubility was tested by mixing small quantities (typically 5.0 mg) of drug (powder) with ACA monomer (typically 100 μL), containing an inhibitor if appropriate (typically 5–10% (w/w), vanillin, Sigma Aldrich, Germany) (Schmid et al., 2022). The blend was visually monitored after 24 h to determine degree of solubility and polymerisation.

### 2.4 Synthesis and physicochemical characterisation of PACA nanoparticles

PEGylated PACA NPs were synthesised by miniemulsion polymerisation. An oil phase consisting of 0.8 g alkyl cyanoacrylate or blend of alkyl cyanoacrylates containing 0–5.0% (w/w) inhibitor, typically vanillin (Sigma Aldrich, Germany) and 0–5% (w/w) co-stabiliser Miglyol 812 (Cremer, USA) was prepared (Schmid et al., 2022). Fluorescent particles for optical imaging were prepared by adding NR668 (modified Nile Red, custom synthesis (Klymchenko et al., 2012), 0.59% (w/w)) to the oil phase. Particles containing an active pharmaceutical ingredient (API) were prepared by adding 5% (w/w) API to the oil phase.

The oil phase was added to an aqueous phase consisting of 0.1 M HCl (12 g) containing Brij L23 (8 mM, Sigma, USA) and Kolliphor HS15 (10.2 mM, Sigma, Germany) and the mixture was immediately sonicated for 3 min on ice (6 × 30 sec intervals, 50% amplitude, Branson Ultrasonics digital sonifier 450, USA). The solution was rotated (15 rpm, SB3 rotator, Stuart, UK) at room temperature overnight before adjusting the pH to 5 using 1 M NaOH. The polymerisation was continued for 5 h at room temperature while rotating. The dispersion was dialysed (Spectra/Por dialysis membrane MWCO 100,000 Da, Spectrum Labs, USA) against water to remove unreacted PEG. The size, polydispersity index (PDI) and the zeta potential of the NPs were measured by dynamic light scattering and laser Doppler Micro-electrophoresis using a Zetasizer Nano ZS (Malvern Instruments, UK) in 10 mM phosphate buffer, pH 7. The reported NP size is the *Z*-average.

Dry weight was determined by drying 3 parallel samples of the particle solution at 50 °C overnight in a ventilated oven. Dry weight is calculated as a percentage, by dividing the dry mass by the solution start mass and an average taken of the 3 parallels. Assuming a density of 1 g/mL NP solution, the dry weight was used to estimate the NP concentration (*e.g.* 1% (w/w) equals 10 mg NPs/mL).

To calculate the amount of encapsulated CBZ (drug fraction), the drug was extracted from the particles by dissolving them in acetone (1:10) and quantified by liquid chromatography coupled to mass spectrometry (LC-MS/MS) as described below. The drug fraction (%), indicating the amount of drug loaded per unit weight of nanoparticles, was calculated by dividing the entrapped drug concentration (mg/mL) by the concentration of NPs in the formulation (mg/mL).

### 2.5 CBZ quantification by LC-MS/MS

CBZ was quantified by LC-MS/MS, using an Agilent 1290 HPLC system coupled to an Agilent 6490 triple quadrupole mass spectrometer. An Ascentis Express C8, 75 × 2.1 mm, 2.7 μm particles size HPLC column with a 5 × 2.1 mm guard column of the same material (Sigma Aldrich), run at 40 °C, was used for sample separation. Mobile phase A was 25 mM formic acid in water and mobile phase B was 100% methanol. The mobile phase gradient was isocratic at 55% B for 1.5 min, then increasing from 55% to 80% B over 1 min, followed by a 1 min washout time and subsequently column re-equilibration. The flow rate was 0.5 mL/min, and 5 μL sample was injected. MS detection was in positive ESI mode (Agilent Jetstream) quantified using multiple reaction monitoring (MRM) mode with the transition *m*/*z* 858.3 to 577.2. The CBZ Na adduct gave the best sensitivity and was therefore chosen as parent ion. Similarly, a hexadeuterated internal standard (catalogue number C046502, Toronto Research Chemicals, Canada) was detected on the 864.4 to 583.2 transition.

### 2.6 Cellular toxicity of PACA NPs

The cell lines used for determining PACA nanoparticle cytotoxicity were Hep G2 and LLC-PK1, cultivated as described in Section 2.1. Cellular toxicity was assessed using the MTT (3-(4,5-dimethylthiazol-2-yl)-2,5-diphenyltetrazolium bromide, Sigma M5655) cell viability assay and the LDH cytotoxicity assay (K311–400, Biovision). Digitonin (Sigma, D141) and Triton-X-100 (Sigma, 93 443) were included as positive controls. Both protocols are a part of the *in vitro* NCL preclinical characterisation cascade; GTA-001 and GTA-002, respectively (Nanotechnology Characterization Laboratory (NCL), 2021b).

#### 2.6.1 MTT cell viability assay

Hep G2 and LLC-PK1 cells were seeded in 96 well plates (Nunc, 3598) with 5 × 10^4^ and 2.5 × 10^4^ cells/well, respectively. After seeding, the cells were incubated overnight (37 °C, 85% relative humidity, 5% CO_2_) to allow cell adhesion. Sample dilution and addition were performed using a Tecan Freedom EVO 200 robotic system. The cells were incubated for 48 h with the different NPs (0.046–300 (600 for 1-pentyl cyanoacrylate (1-PCA), 2-heptyl cyanoacrylate (2-HPCA) and 3,3-dimethylbutyl cyanoacrylate (3,3-DMBCA)) μg/mL). The MTT assay was performed using a fully automated method on a Beckman FX^P^ robotic system, integrated with CO_2_ incubator, plate reader and a SCARA arm. In short, cell medium was aspirated and exchanged with 250 μL of medium containing a final concentration of 1 mg MTT/mL. The incubation was continued for 3 h at 37 °C to allow formation of the formazan-particles, after which medium was discarded and formazan particles were dissolved in DMSO with 0.1 M glycine and 0.1 M NaCl. The absorbance was read by a plate reader (SpectraMax I3X, Molecular Devices) at 570 nm, and background from absorbance at 650 nm was subtracted.

#### 2.6.2 LDH cytotoxicity assay

As part of a fully automated procedure, LDH release was quantitated in the supernatant collected from the MTT well plates described above. After NP incubation, 50 μL of supernatant was transferred to a new well plate, before reaction mixture (prepared according to manufacturer's protocol) was added to each well. The mixture was incubated for 20 min at room temperature in the dark. The absorbance was read by a plate reader (SpectraMax I3X, Molecular Devices) at 490 nm, and background from absorbance at 680 nm was subtracted.

### 2.7 *In vivo* toxicity of PACA NPs

#### 2.7.1 Animal model

Female Sprague Dawley rats (Janvier, France) were purchased at 10 weeks old and used at body weight of approx. 300 g. The animals were housed in individually ventilated cages and provided with clean food (RM1(E), SDS, UK) and sterile water *ad libitum*. The housing conditions were free of specific pathogens according to recommendations from the Federation of European Laboratory Animal Science Association, with controlled temperatures between 19 and 22 °C, relative humidity between 50 and 60% and 12 h light and dark cycles. All animals were acclimatised for one week before starting the experiment. All experimental procedures involving laboratory animals were performed in compliance with protocols approved by the Norwegian Food Safety Authorities, according to EU directive 2010/63/EU on the protection of animals used for scientific purposes, and in accordance with the 3 R's principle.

#### 2.7.2 Toxicity study design

Toxicity effects following a single i.p. injection of PEBCA or PEHCA NPs (75 mg NPs/kg) were studied in healthy rats (*n* = 4 per timepoint). This dose was chosen based on previous work, where CBZ showed therapeutic efficacy with a dose of 7.5 mg/kg (Snipstad et al., 2021), and taking into account that its fraction in PEBCA-CBZ NPs is approx. 10% (w/w). The injection solutions were prepared directly before use by diluting the NP particle stock solutions in 0.9% NaCl. An equivalent volume of 0.9% NaCl was used as negative control. Before injection, animals were anesthetised with isoflurane, and morphine analgesia (1 mg/kg) was administered subcutaneously (s.c.) directly after NP injection.

All rats were monitored regularly for health status and body weight. Blood and tissue samples were harvested at 4 h, 3 days, 10 days and 16 days post-injection after exsanguination under full anaesthesia. Selected organs (brain, liver, spleen, kidneys, lung and heart) were weighed immediately after dissection.

#### 2.7.3 Histopathology

Organs (kidneys, liver and an approx. 2 × 1 cm section of the parietal peritoneum, excised from near the i.p. injection site) were fixed for a minimum of 2 days in 4% neutral buffered formaldehyde. The samples were then embedded in paraffin, before 4 μm sections (Leica Tissue processor) were stained with hematoxylin and erythrosin (HE) and imaged by a veterinary pathologist to evaluate the state of the tissue after NP injection.

#### 2.7.4 Blood samples

Blood samples were taken during anaesthesia by terminal cardiac puncture in tubes prefilled with EDTA (Sarstedt Microvette® 500 μL, K3 EDTA) for blood haematology and in tubes with Clotting Activator (Sarstedt Microvette® 500 μL, Clotting Activator/Serum) for determination of clinical chemistry parameters. The EDTA tubes were gently inverted 8–10 times to ensure proper mixing with the coagulant, before being stored at 4 °C until they were analysed for haematological parameters within 24 h. The analysis included a differential count for the number of white blood cells (total WBC and subtypes), platelets and red blood cells (RBC). Additional analytical parameters included the concentration of haemoglobin (HGB), haematocrit (HCT), mean red blood cell volume (MCV), mean haemoglobin concentration in red blood cells (MCHC) and red blood cell distribution width (RDW).

The Clotting Activator serum tubes were left to clot in an upright position at room temperature for 1 h, before the serum was separated from the blood cells by centrifugation (5 min, 10,000 rcf, room temperature). The serum was transferred to new, empty tubes, and kept frozen (−80 °C) until clinical chemistry analysis. The analysed parameters included alanine aminotransferase (ALT), amylase (Amy), alkaline phosphatase (AP), aspartate aminotransferase (AST) and creatin kinase (CK), total bilirubin, creatinine, urea, albumin, total protein, glucose, inorganic phosphate, potassium, calcium, chloride and sodium.

### 2.8 Statistical analysis

The obtained results are presented as mean ± standard deviation (SD). One-way analysis of variance (ANOVA) with post-hoc comparisons by Dunnett's *t*-test was applied on animal weight data, organ-to-brain weight data and blood sample data. In all instances, *p* values ≤0.05 were considered statistically significant. The statistical analyses were performed using GraphPad Prism (version 9.2.0 for Windows, GraphPad Software, La Jolla, California, US).

## 3 Results

### 3.1 *In vitro* cytotoxicity of alcohols

**Fig. 1:**
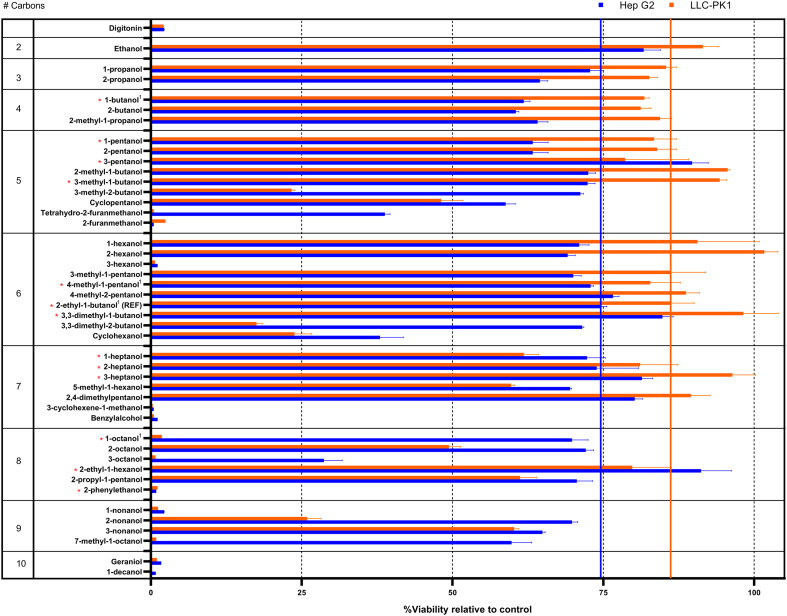
Cellular viability (% relative to untreated control cells) after 3% (v/v) alcohol exposure in Hep G2 (blue) and LLC-PK1 (orange) cell lines. Alcohols are arranged by their number of carbon atoms (left column). Vertical lines indicate mean viability levels for the reference alcohol 2-ethyl-1-butanol, and alcohols chosen for further characterisation are marked with red asterisks. ^1^indicates alcohols corresponding to commercially available ACAs. Digitonin was included as positive control. (For interpretation of the references to colour in this figure legend, the reader is referred to the web version of this article.)

A high-throughput cytotoxicity screening of the selected 44 alcohols was performed in Hep G2 (liver carcinoma) and LLC-PK1 (kidney epithelial) cell lines. Concentration levels below the highest alcohol concentration level (3% (v/v)) induced limited or no toxicity in both cell lines, and few differences could be observed between the different alcohols (data not shown). As a result, no half-maximal inhibitory concentrations (IC_50_ values) could be calculated. Instead, the % cell viability after 3% (v/v) alcohol exposure relative to untreated cells is shown in Fig. 1. The alcohols are arranged by their number of carbon atoms to better understand the varying toxicity levels.

Large differences in cytotoxicity could be found for the various alcohols tested, also between the different cell lines. A trend points towards increasing toxicity with increasing number of carbons and increasing complexity/branching of the carbon chain. Generally, higher viability levels could be observed for the LLC-PK1 cell line, especially for alcohols with <8 carbon atoms. In the LLC-PK1 cell line, 9 alcohols showed lowered cell toxicity compared to the 2-ethyl-butanol reference (seen to the right of the orange vertical line in Fig. 1), with 2-hexanol, 3,3-dimethyl-1-butanol and 3-heptanol being the three best candidates. 7 Alcohols showed lowered cell toxicity in the Hep G2 cell line (seen to the right of the blue vertical line in Fig. 1), with 2-ethyl-1-hexanol, 3-pentanol and 3,3-dimethyl-1-butanol as lead candidates.

ACAs based upon twelve alcohols were chosen, obtained and used in NP synthesis based on their toxicity profile (marked with a red asterisk in Fig. 1). These included the alcohols corresponding to the four commercially available and routinely used (for nanoparticle applications) ACA monomers BCA, 2-EBCA, 4-MPCA and OCA (1-butanol, 2-ethyl-1-butanol, 4-methyl-pentanol and 1-octanol, respectively). The selected alcohols showed reduced toxicity in at least one cell line when compared to the commercially available controls. In addition, regardless of its high toxicity, 2-phenylethanol was chosen as a synthetic target of interest, as it has structural features that could enable an improved range of active ingredients to be encapsulated in a novel PACA. This was especially based on its potential to enhance API solubility through π-π stacking interactions. Neopentanol, a solid alcohol not tested in the alcohol screening, was also included in the following characterisation based on its theoretical potential to enhance API solubility and ready availability as an ACA monomer.

### 3.2 Drug compatibility

A solubility screening was performed to evaluate the compatibility between different monomers and selected APIs: the microtubule inhibiting taxanes cabazitaxel (CBZ) and docetaxel, the alkylating platinum-based drug oxaliplatin and the antibiotic mupirocin. These are all hydrophobic drugs with limited bioavailability, where nanoencapsulation potentially may increase their therapeutic efficacy. The results showed large variations in drug solubility between different monomers (Table 2). As anticipated, the inclusion of the 2-PECA/BCA blend broadened the solubility range of APIs, as shown by the dissolution of oxaliplatin, which hence enables its NP encapsulation in PACA. None of the other monomers tested were able to dissolve this API, which supports the theory of increased solubility by the 2-PECA/API π-π interactions. The variation in API solubility in monomers with alkyl chains of different lengths and substitution pattern was exemplified by the solubility of mupirocin, which was insoluble in the controls (BCA, 2-EBCA and OCA), but soluble in 3-PCA and 2-EHCA. CBZ was soluble in all ACAs tested and was therefore chosen as model drug for physicochemical characterisation of NPs in the following screening step. CBZ was additionally shown to be soluble in the 4-MPCA monomer (not shown), but 4-MPCA was not included in the broader drug compatibility screen due to material limitations.

**Table 2:** Results from the solubility screening of PACA monomers and selected hydrophobic drugs.

Monomer(s)	Corresponding alcohol(s)	Solubility			
		*Cabazitaxel*	*Docetaxel*	*Oxaliplatin*	*Mupirocin*
BCA^a^	1-butanol	**Soluble**	**Soluble**	Insoluble	Insoluble
1-PCA	1-pentanol	**Soluble**	Insoluble	Insoluble	Insoluble
3-PCA	3-pentanol	**Soluble**	**Soluble**	Insoluble	**Soluble**
3-MBCA	3-methyl-1-butanol	**Soluble**	**Soluble**	Insoluble	Insoluble
1:2 NPCA:BCA	Neopentanol/1-butanol	**Soluble**	**Soluble**	Insoluble	Insoluble
2-EBCA^a^	2-ethyl-1-butanol	**Soluble**	Partially	Insoluble	Insoluble
3,3-DMBCA	3,3-dimethyl-1-butanol	**Soluble**	Insoluble	Insoluble	Insoluble
1-HPCA	1-heptanol	**Soluble**	**Soluble**	Insoluble	Insoluble
2-HPCA	2-heptanol	**Soluble**	Insoluble	Insoluble	**Soluble**
3-HPCA	3-heptanol	**Soluble**	Insoluble	Insoluble	Partially
OCA^a^	1-octanol	**Soluble**	**Soluble**	Insoluble	Insoluble
2-EHCA	2-ethyl-1-hexanol	**Soluble**	Insoluble	Insoluble	Partially
1:2 (2-PECA):BCA	2-phenylethanol/1-butanol	**Soluble**	**Soluble**	**Soluble**	Insoluble

a Indicates commercially available ACAs.

As the lead candidates from alcohol and NP cytotoxicity screening became clear, the solubility of the histone deacetylase (HDAC) inhibitor belinostat was tested in the novel 1-HPCA and 2-EHCA monomers, in addition to the 2-EBCA control. Due to limited material, belinostat was only tested in these three ACAs. Interestingly, the results showed that belinostat was soluble in both 1-HPCA and 2-EHCA, but not in the 2-EBCA control.

### 3.3 Physicochemical properties of PACA NPs

**Fig. 2:**
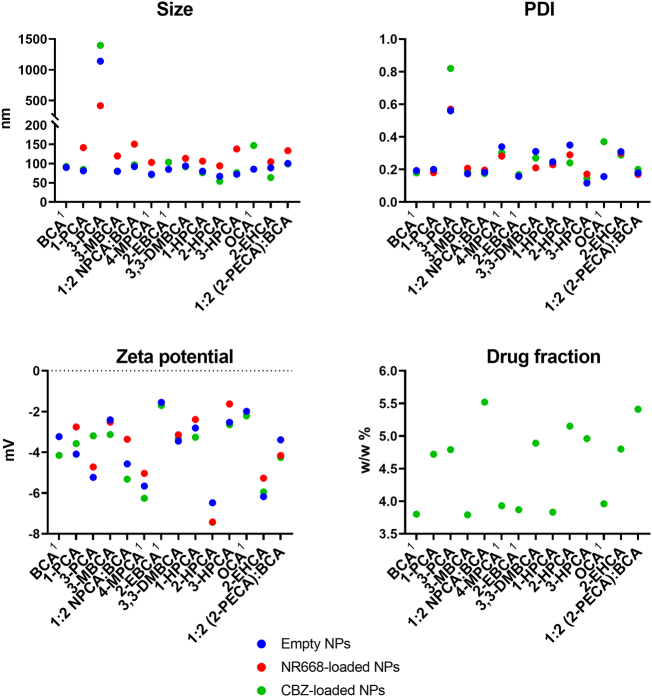
Physicochemical properties of PACA NPs. The upper and middle panels show size, PDI, zeta potential and CBZ drug fraction for all NP batches. The lower panel shows scatter plots of size, PDI and zeta potential. ^1^ indicates commercially available ACAs.

Empty particles were synthesised using the same miniemulsion polymerisation protocol, originally standardised for polymerisation of the reference monomers BCA, 2-EBCA, 4-MPCA and OCA. The physicochemical properties of the synthesised NPs are summarised in Fig. 2. Empty particles had a hydrodynamic diameter ranging between 72 and 112 nm with a polydispersity index (PDI) below 0.35. The surface charge (zeta potential) of the particles was slightly negative, ranging from −6.5 to −1.6 mV. Particles made from 3-PCA are the only exception, showing a larger *Z*-average (1139 nm) and a broader size distribution (PDI = 0.56). This trend for increase size when using 3-PCA was also seen for both the NR668 and CBZ loaded particles, and indicates non-colloidal suspensions after failed miniemulsion polymerisation. Based on this, 3-PCA was not chosen for further development and use.

The effect of encapsulation on size, size distribution and zeta potential was studied using CBZ and the fluorescent dye (NR668). NR668 is a hydrophobic model drug enabling optical investigation of PACA NP, *e.g.* in *in vitro* intracellular studies and *in vivo* biodistribution studies (Sulheim et al., 2016; Pandya et al., 2021). Encapsulation of the fluorophore resulted in a slight, but not significant, increase in particle size, while no change in size could be observed after encapsulation of CBZ. The PDI and zeta potential also remained unchanged.

The CBZ drug fraction was in the range of 3.8–5.5% for all NP batches. The two highest levels were found in formulations based on blends of a solid ACA monomer and BCA: NPCA/BCA and 2-PECA/BCA, with drug fractions of 5.4 and 5.5%, respectively.

### 3.4 *In vitro* cytotoxicity of NPs

Based on the alcohol toxicity profile and the NP physicochemical properties, 13 different NPs were chosen for toxicity screening in cells. This study was performed using the standardised test regime established by NCL for toxicity profiling of nanomaterials (GTA-001 and GTA-002 protocols) (Nanotechnology Characterization Laboratory (NCL), 2021b). NPs were added to Hep G2 liver cells and LLC-PK1 kidney cells, and the viability was evaluated after 48 h of incubation by MTT and LDH assays. The MTT assay gives an estimate of the metabolic activity of the cells by measuring the conversion of the MTT reagent to a coloured product. The LDH assay, on the other hand, quantitates the release of the cytoplasmic enzyme LDH after breach of cell membrane integrity.

**Fig. 3:**
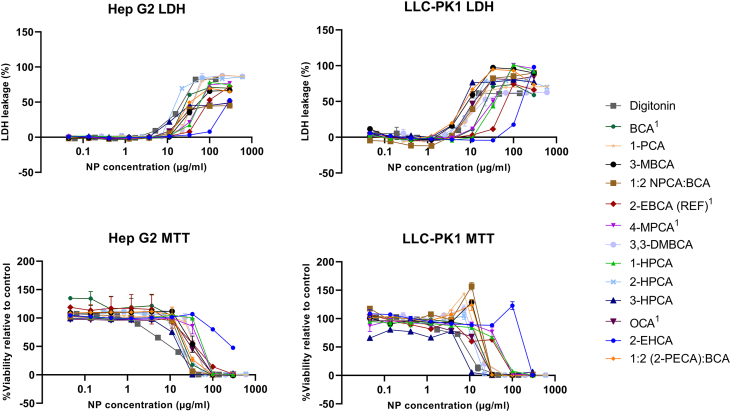
Results from LDH cytotoxicity (upper panel) and MTT viability (lower panel) assays in Hep G2 and LLC-PK1 cell lines. Digitonin was included as positive control. ^1^ indicates commercially available ACAs.

The results are shown in Fig. 3, and the estimated IC_50_ values are listed in Table 3. Polymer composition was found to clearly affect the cytotoxicity, with PEHCA NPs prepared from the 2-EHCA monomer, being the least toxic in both cell lines, based on both MTT and LDH analysis. PEHCA NPs were also the only NPs to outperform the reference PEBCA NPs in both cell lines by both assays. The promising PEHCA results were confirmed in a repeated study (data not shown). Interestingly, the resulting IC_50_ values demonstrate large variations between the cell lines, especially when looking at the LDH results. A smaller variation, and generally lower IC_50_ values, seen for the MTT results can be explained by NP-cell interactions causing cell death without membrane damage. 3-HPCA NPs outperform the PEBCA reference particles in both cell lines when comparing the MTT results, while they show higher amounts of LDH release. This indicates that other cellular mechanisms are involved in the cell's reaction to these particles than to PEBCA NPs. Based on the *in vitro* results, only PEHCA NPs were chosen for further characterisation *in vivo*.

**Table 3:** Calculated IC_50_ values for PACA NPs in Hep G2 and LLC-PK1 cell lines, by both LDH and MTT assays. PACA NPs are arranged by decreasing IC_50_ values in Hep G2 cells measured by the LDH assay. Digitonin was included as positive control.

	IC_50_ values (μg/mL)			
	LDH		MTT	
	*Hep G2*	*LLC-PK1*	*Hep G2*	*LLC-PK1*
Digitonin	022.6	029.6	009.3	007.3
2-EHCA	286.0	135.8	274.8	>300.0
1:2 NPCA:BCA	219.7	015.0	019.7	027.1
OCA^a^	186.7	014.0	032.1	013.7
3-HPCA	160.9	008.3	013.5	002.7
2-EBCA (REF)^a^	116.8	083.3	038.4	025.5
3-MBCA	078.4	009.0	033.7	026.6
4-MPCA^a^	066.5	032.1	045.8	044.3
1-HPCA	065.2	034.0	078.7	041.8
1:2 (2-PECA):BCA	060.8	007.7	031.1	026.3
BCA^a^	046.3	040.0	029.1	015.0
PCA	042.3	032.2	035.8	020.4
3.3-DMBCA	040.0	059.3	024.1	020.3
2-HPCA	017.5	014.0	021.5	018.6

a Indicates commercially available ACAs and (REF) indicates reference PEBCA NPs (2-EBCA monomer).

**Fig. 4:**
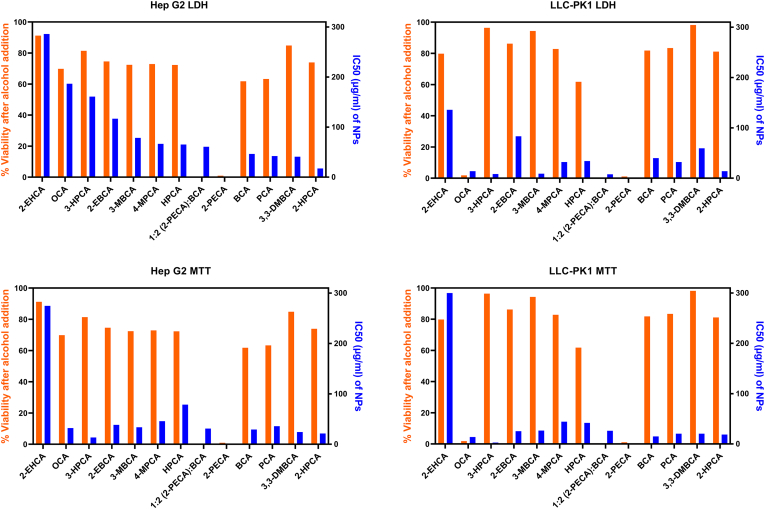
Comparison of viability (%) after alcohol addition (orange, left axes) and IC_50_ values after NP addition (blue, right axes) in Hep G2 and LLC-PK1 cell lines in both LDH and MTT assays. Note that the 2-PECA:BCA blend was tested for NP toxicity only, and the toxicity of the corresponding alcohol blend is therefore not included. For 2-PECA, only the toxicity of the corresponding 2-phenylethanol alcohol was tested. (For interpretation of the references to colour in this figure legend, the reader is referred to the web version of this article.)

To explore the relationship between the toxic responses of the free alcohol degradation products and the NPs, a correlation study was performed. Fig. 4 shows a graphic representation of the viability after addition of 3% alcohol (in orange), and the IC_50_ values obtained from the NP cytotoxicity screening (in blue). In general, a large variation could be observed between the different NP IC_50_ values, while the alcohol toxicity study typically showed viability at more comparable levels between 60 and 100%. No correlation between the two parameters could be found (Fig. S2). Some observations are, however, worth mentioning. 2-PECA, showing the highest level of cytotoxicity in the alcohol screening, induced relatively high toxicity levels (IC_50_ values <61 μg/mL) in both cell lines even after being mixed with BCA to form NPs, supporting the results from the alcohol screening. Surprisingly, the toxicity of pure BCA NPs was higher than that of the *co*-polymeric PECA/BCA NPs, where BCA is blended with the 2-PECA monomer which can release the supposedly very toxic 2-ethylphenylalcohol on hydrolysis. We propose that this difference results from inclusion of PECA reducing the hydrolysis rate of the corresponding co-polymers *vs* that of the pure PBCA polymer, reducing the alcohol concentrations in the vicinity of the cells. A clear toxic effect was observed for both 1-octanol and the corresponding POCA NPs, but only in the LLC-PK1 cell line. 3-heptanol was one of the lead candidates from the alcohol screening by showing very low toxicity responses in both cell lines (>80% viability). NPs prepared from the corresponding 3-HPCA monomer were surprisingly shown to induce high cytotoxic effects, especially in the LLC-PK1 cell line (IC_50_ values <10 μg/mL). The low cytotoxic effects of 2-PEHCA NPs stand out, and this trend was also seen in the alcohol screening, with viability levels of 91.2 and 79.8% in Hep G2 and LLC-PK1 cells, respectively.

### 3.5 *In vivo* toxicity of PACA NPs

As a last step in the pre-clinical toxicity screening cascade, an *in vivo* toxicity screening was performed. Two different PACA NPs were chosen for the study: the go-to commercially alternative PEBCA, and the PEHCA lead candidate from the cytotoxicity screen.

#### 3.5.1 Clinical signs, animal weight and organ weight evaluation

**Fig. 5:**
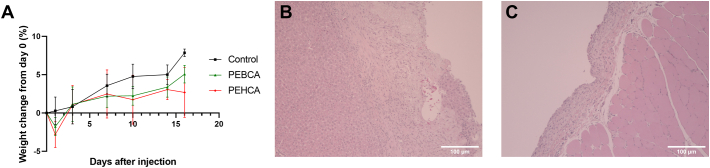
Results from the rat toxicity study. A) Rat body weight change after NP injection. Number of animals at the timepoints are reduced as animals are euthanised: Day 1 and 3 (12 PEBCA, 12 PEHCA, 6 control); day 7 and 10 (8 PEBCA, 8 PEHCA, 4 control); day 14 and 16 (4 PEBCA, 4 PECHA, 2 control). B-C) Photomicrographs of tissue sections (HE staining) showing representative effects observed after i.p. NP injection. Increased amounts of fibrous connective tissue could be seen both in liver (B) and peritoneal (C) tissue.

General and pathological symptoms of animals were investigated during and after i.p. administration of PACA NPs. At the studied concentration, injection of both PEBCA and PEHCA NPs resulted in a rapid and significant decrease in body weight compared to the saline control 24 h after injection (*p* values: 0.0254 and 0.0009, respectively) (Fig. 5A). This effect is probably a result of abdominal pain and limited food and water intake, as we observed signs of abdominal constriction (lateral contortion of the flank abdominal muscles) in the NP treated animals during the first hours after injection. This is a common response to irritations in the peritoneal cavity after i.p. injection (Collier et al., 1968). After this observation, all i.p. injections were performed in combination with s.c. injection of morphine analgesia. All animals had regained their normal behaviour at the health monitoring the following morning (14 h after injection) and recovered their initial body weight by day 3 after injection. At this time point, the net weight gain in both NP groups was comparable to the control group. The NP injected animals continued to increase their body weight, and no animals were euthanised before the planned end points at 4 h, 3 days, 10 days and 16 days.

After dissection, the brain, liver, spleen, heart and kidneys were weighed, and organ-to-brain weight ratios were calculated. This is a helpful measure when the terminal body weights are affected by the test article and can be used to normalise the organ weight data in cases with large inter-animal variability (Nirogi et al., 2014; Bailey et al., 2004; Sellers et al., 2007). Additionally, the organ-to-brain weight calculation normalises for animal weight gain over time, allowing for comparison of results from different study time points. The calculated organ-to-brain weight ratios showed no significant differences between the treated groups and the control, except one single observation in elevated spleen-to-brain weight ratios in the PEHCA group 3 days post-injection (Fig. S3). Splenic enlargement is mostly a sign of increased immune activity, including increased antigen clearance, antibody production and number of reticuloendothelial cells contained within the spleen. In other cases, liver disease and congestion can lead to increased venous pressure causing enlargement of the spleen (Chapman et al., 2021).

### 3.6 Biochemical parameters after NP injection

The effect of administration of PACA NPs on haematological and biochemical parameters was determined using blood assays. The results are listed in Tables S2 and S3, respectively. Few statistically significant differences could be observed between animals injected with NPs and the saline control, and all responses were normalised at the last sampling time point 16 days post-injection. An acute and transient immune response was observed in both treatment groups, with an increased number of innate immune cells, including neutrophils and monocytes. The neutrophil response was shown to be stronger in PEHCA treated animals compared to the PEBCA group.

A response was also seen for the red blood cells, with a significant increase in total count, haemoglobin concentration and haematocrit (the volume of red blood cells per volume of whole blood). In the PEBCA treatment group, this effect was most prominent 10 days post-injection, while a weaker response was seen in the PEHCA group, but at both the 4 h and 10-day time points. A decrease in the platelet count could be observed for both treatment groups, especially at the 10-day time point. The effect was more prominent in the PEBCA group compared to that of PEHCA, but the response was normalised at the 16-day time points for both groups.

Clinical chemistry analysis showed very few differences between the NP treated groups and the saline control group. The observations point towards minor changes in the electrolyte balance, including initial decreases in sodium and chloride concentrations in both treatment groups. An initial and transient increase in inorganic phosphate was seen, especially for the PEHCA group. Decreases in albumin and creatinine serum concentrations, and minor increases in liver ALT enzymes were observed in both NP groups.

#### 3.6.1 Histopathology

Only mild reactions were observed in tissue sections prepared from both PEBCA and PEHCA groups. Liver evaluation showed incipient signs of focal necrosis, fibrosis and mild inflammation on the organ surface (Fig. 5B). Liver cells were evaluated as normal. Mild inflammation was also seen on the kidney surface, and on the peritoneum wall (Fig. 5C). All observations were evaluated as of no clinical significance, but as effects of the local i.p. injection.

## 4 Discussion

Novel PACA materials can allow for synthesis of NPs with optimised drug loading capacities, drug compatibility and toxicity profile. The process of finding such new materials requires substantial screening and characterisation, including toxicity analysis in cell lines and animal models.

In this study, the toxicity of 44 different alcohols was quantitated in two relevant cell lines, resulting in the selection of 13 alcohols. The 13 corresponding ACA monomers, and a 14th, NPCA included as the ACA only, as readily available (neopentyl alcohol is solid and could not be include in the alcohol toxicity screen) were chosen for further work. The ACAs were studied for their capacity to solubilise relevant APIs and were used to prepare nanoparticles with or without encapsulating model drugs. After further *in vitro* toxicity analysis, 2-PEHCA was taken forward to *in vivo* toxicity studies.

### 4.1 Toxicity

The studied alcohols were chosen based on a number of criteria. Firstly, the selection was limited to primary and secondary alcohols, due to anticipated difficulties obtaining cyanoacrylate esters of tertiary or very sterically hindered alcohols. Secondly, low molecular weight alcohols with carbon numbers below 4 were excluded due to known reactivity of such ACA monomers (compared to those with ≥4 carbon), whereas upper molecular weight limits were chosen based upon desire to have liquid ACAs for use in miniemulsion polymerisation particle synthesis (stopping at C = 10). Then for each carbon length, all isomers that were readily and cost effectively commercially available were included (*e.g.* we would be able to prepare/obtain the corresponding ACA without difficulty sourcing the alcohol). Racemic alcohols were used where relevant. Some simple cyclic alcohols (cyclohexanol and cyclopentanol) were included for comparison. Finally, a selection of unsaturated and aromatic alcohols was screened to test the hypothesis that ACA based on such alcohols could give enhanced API solubility through π-π stacking interactions (*e.g.* geraniol, 2-furanmethanol, benzyl alcohol and 2-ethylphenyl alcohol). For such materials, it was not a concern if the resulting ACA was likely to be a solid, as the material could be blended into a liquid ACA for NP synthesis.

As a first step in the toxicity screening, the cellular viability after 24 h exposure of 3% (v/v) alcohol was measured. The 3% alcohol dose corresponds to a concentration of approx. 30 mg/mL (assuming an alcohol density of approx.1 g/mL). In preclinical treatment studies, the PACA NP dose is often between 100 and 200 mg/kg (Fusser et al., 2019; Snipstad et al., 2017; Snipstad et al., 2021), leading to blood concentrations of approx. 1.7–3.4 mg NP/mL blood (in 20 g mice with 1.17 mL total blood volume (National Centre for the Replacement, 2022)). These NP concentrations are not able to release 30 mg/mL of alcohols and the release of alcohol occurs over a prolonged time and not acutely. The alcohol concentration tested is therefore high compared to clinically relevant alcohol levels in blood, but is of high relevance for tissues where NPs accumulate, *e.g.* in the liver (Fusser et al., 2019; Snipstad et al., 2017). The results from the liver cell line Hep G2 are therefore emphasised in our work. Based on this, the toxic effect of potential accumulation of alcohol degradation products is an important study parameter in the evaluation of novel ACA materials for use in biomedical applications. Materials forming alcohols with high toxicity should not be used and should therefore be excluded from further evaluation. Whilst it does not account for the kinetics of the ester hydrolysis required to release the alcohol, the alcohol screening step is also a simple and practical way to screen a large number of compounds without synthesising or buying expensive ACA monomers. In this way, a large portion of alcohol alternatives showing high toxicity can be excluded from more laborious experiments in following screening steps.

In a biological system, PACA can degrade before, as well as after cellular uptake, resulting in both extra- and intracellular alcohol exposure. Alcohol molecules are known to interact with cellular and subcellular membranes and change the membrane's structural and/or dynamical properties (Kondela et al., 2017). These changes can influence the conformation of proteins or other structures embedded in the membrane and influence the cellular function and survival. Alcohol-membrane interactions can therefore be detrimental to cells, and this effect is shown to be chain length dependent, as the larger hydrophobic regions on long-chain alcohols favourably interact with the lipid bilayer hydrocarbon chains and hence enable membrane penetration (Patočka and Kuča, 2012). Other potentially toxic effects caused by intracellular alcohol metabolism include free radical formation and oxidation to toxic aldehydes and acids, but these processes are not fully understood (Patočka and Kuča, 2012; Strubelt et al., 1999).

The observed differences in cellular toxicity after alcohol exposure can be linked to the structure of the alcohol, as an increasing number of carbons and increasing complexity/branching of the carbon chain generally seem to induce higher toxicity responses in both cell lines. This is not in line with the general perception that alcohol toxicity decreases with increasing carbon chain length and that higher aliphatic saturated alcohols with seven or more carbon atoms are toxicologically insignificant (Patočka and Kuča, 2012). It is though important to point out that our observations are based on cell exposure studies, and not on whole animal exposure route studies (*e.g. via* ingestion, inhalation or consumption) that more accurately mimic true conditions. Longer alcohols have a structure resembling that of fatty acids and waxes and have a close relation to different biological compounds. They also show a decrease in polarity compared to that of shorter aliphatic alcohols, indicating a decreasing water solubility and concurrent higher lipophilicity.

Interestingly, some trends in our dataset indicate that certain alcohol chemistries induce specific toxic responses, not in line with the overarching trends. 2-ethyl-1-hexanol (the alcohol corresponding to PEHCA NPs) is one such example, with a low toxic effect compared to that of alcohols with the same number of carbons (octanol and 2-propyl-1-pentanol). These observations are in line with observations from Sulheim et al., where degradation products from PBCA and POCA NPs showed much higher toxicity in Hep G2 cells than degraded PEBCA NPs (Sulheim et al., 2017). This could not be easily explained by the size of the monomer side chain, as the 2-EBCA has an intermediate molecular weight (BCA < 2-EBCA < OCA) but could rather be linked to the branched composition of the side chain.

The second step in the toxicity screening was to compare the cytotoxic effects of NPs prepared from the different monomers. Large variations could be seen also here, and no obvious trends are found to explain the phenomenon. The lack of correlation found between the two screening experiments indicates that the NP toxicity is not directly linked to the alcohol toxicity alone, but also caused by additional factors. These may include NP size, surface charge, molecular weight, cellular uptake and NP degradation rate (Sulheim et al., 2017; Sukhanova et al., 2018; Egbuna et al., 2021).

PEHCA NPs clearly outperformed NPs prepared from the current market leaders BCA, 2-EBCA, 4-MPCA and OCA in the cellular studies. While this can be explained by the low toxicity profile of the alcohol degradation product alone, it cannot be ruled out that hydrolysis rate also has a role in the observed reduced toxicity. The higher toxicity levels seen for the other NPs tested, including ones with alcohol toxicity profiles comparable to that of PEHCA NPs, can be a result of degradation rates leading to higher local concentrations of degradation products and hence a higher toxic effect on the nearby cells. Differences in NP-cell interactions and cellular uptake rates and mechanisms can also contribute to the observed effects. Additionally, cell line-dependent interaction mechanisms are of great importance for NP cytotoxicity (Sulheim et al., 2017). Differences in uptake rates and uptake pathways may lead to different cellular responses and tolerability, which shows the importance of studying toxicity levels in several relevant cell lines. More information on NP degradation and cellular uptake are, however, required to confirm these hypotheses, and this is therefore the subject of ongoing studies in our institute.

Cell culture models are regularly used to study NP toxicity. Such *in vitro* assays are assumed to adequately simulate the *in vivo* physiologic environment but will never truly represent the complexity seen *in vivo*. The time- and cost-effectiveness of the *in vitro* evaluation is, however, valuable as a screening step prior to the more labour-intensive *in vivo* studies. Although the *in vivo* experiment was performed with ip administration, the performed *in vitro* assays are relevant, as a newly performed biodistribution assessment of both cabazitaxel and PEBCA NP degradation products after ip injection of PEBCA-cabazitaxel NPs showed, that both the drug and the NP material enters the blood stream and accumulates in the liver (unpublished results). Systemic toxicity is therefore highly relevant for ip therapies, as it is for intravenous therapies. Our results show a low correlation between toxicity levels of PEBCA and PEHCA NPs *in vitro vs in vivo*, as the *in vivo* study showed that the two NP batches induce comparable toxic responses. The large difference in cytotoxicity *seen in vitro* was neutralised by systemic effects, potentially including biodistribution, clearance and NP degradation rates. Differences in the NP's interaction with proteins and other components in blood and/or the peritoneal fluid influence the formation of the NP corona – a factor decisive for the biological and toxicological outcome of NP interaction with a biological system (Nguyen and Lee, 2017; Kopac, 2021; Yordanov et al., 2016).

In more detail, the results from the *in vivo* experiment in rats showed that the animal groups for both NPs quickly regained their weight after an initial weight loss and continued to gain weight throughout the study period. Similarly, analysis of organ weight data showed few differences from control, with splenic enlargement 3 days after PEHCA injection as the only significant observation for one animal. Both NPs were found to cause significant increases in leucocyte counts, with PEHCA inducing a stronger response compared to that of PEBCA. However, both were transient and expected responses caused by the innate immune system, as phagocytic immune cells have specific roles in clearance of foreign material like nanoparticles, bacteria and viruses (Boraschi et al., 2017; de la Harpe et al., 2019).

For erythrocytes, a minor decrease in cell count and haematocrit (day 3) was followed by a significant increase (day 10) for both NP groups. This can indicate a NP-induced initial haemolysis, with a resulting over-production of erythrocytes by the bone marrow. Increased levels of inorganic phosphate, seen at the 4-h time point for both NPs, support this theory. Haemolysis releases intracellular phosphate and thus elevates serum phosphate concentrations (Bansal, 1990). Lowered platelet counts signalise elevated platelet disruption and/or reduced production. NP-platelet interactions can affect platelet function in several ways, including platelet adhesion, activation and aggregation, *e.g.* by causing hypercoagulation (de la Harpe et al., 2019). NPs can also cause bone marrow suppression leading to decreased production of platelets and red and white blood cells. As no decrease in leucocytes could be seen in our study, this theory is not supported (Dadachova, 2012).

Minor changes in electrolyte, urea and creatinine levels may indicate instability in the fluid homeostasis, especially when seen in combination with the weight reduction that was observed the first few days after NP injection. Limited food- and water-intake can largely influence these parameters (Stanhewicz and Larry Kenney, 2015; Feehally and Khosravi, 2015). The lowered albumin levels in serum may be an indication of liver damage, as albumin is synthesised in the liver. Increasing serum levels of the liver ALT enzyme, yet not significantly different from the control, may also point towards liver damage. Histopathological evaluation of liver tissue did, however, not indicate any signs of liver cell pathology. Importantly, at the last day of follow-up (day 16), no blood parameters were significantly different from the control, indicating that all the observed effects were normalised.

Overall, the results show that the novel PEHCA NP system has potential as nanomedicine, giving comparable toxic responses as the go-to commercial PEBCA NP alternative. The somewhat surprising *in vivo* effects of PEHCA injection, not reproducing the outperformance seen *in vitro*, can give rise to thoughts on whether potential surprising *in vivo* effects may be observed also with other novel PACAs not included in the *in vivo* experiment. An example is NPs made from 1-HPCA monomers, showing an improved *in vitro* toxicity profile compared to that of PEBCA in both Hep G2 and LLC-PK1 cell lines when analysed by the MTT assay. This monomer is therefore an interesting candidate for future follow-up studies.

### 4.2 Drug compatibility

Encapsulation of drugs in PACA NP particles can be achieved by sorption or entrapment, and the method chosen usually affects several parameters, including entrapment efficiency and release characteristics of the API (Krauel et al., 2004). For PACA entrapment encapsulation techniques like miniemulsion polymerisation, a drug should be compatible with the monomer. Firstly, it should not trigger premature polymerisation of the monomer, and secondly the degree of drug solubility with the ACA monomer influences the degree of encapsulation possible for the drug. A high loading theoretically allows for high drug dose at lowest possible particle material amount, thus minimising the potential for side effects arising from the nanoparticle delivery system. This is especially important when encapsulating hydrophobic small molecule drugs with high dose requirements or a narrow therapeutic window. Solubilisation (without triggering polymerisation of the monomer) is a simple indicator that encapsulation of a particular drug can be achieved, whilst using only extremely small amount of drug and polymer.

Our results show large variations in drug solubility between the different monomers tested, including cases where only one or two novel monomers, and none of the commercially available alternatives were drug compatible. The drugs chosen, all had good compatibility with the monomers tested, and did not trigger premature polymerisation of the monomer. This illustrates the effect of the different ACA monomer chemistries on drug solubility and emphasises the advantage of having an array of ACA monomers to choose between in the process of encapsulation design for new APIs. Extending the available ACA toolbox can increase the use of PACA NPs by enabling encapsulation of formerly non-compatible drugs and newly developed compounds, especially hydrophobic small molecular drugs. Additionally, it can give flexibility to tailor drug release properties, tune the physicochemical properties of PACA NPs and hence reduce their toxicity. Up to now, PACA NPs have mostly been used in cancer drug delivery and other applications where low therapeutic doses are needed. A reduced material toxicity can extend the use of PACA NPs towards treatment regimens where higher therapeutic doses or dosing over long time are needed, formerly limited by the toxicity of the NPs.

### 4.3 NP physicochemical properties

The results from physicochemical testing of the NP batches show the robustness of the PACA miniemulsion polymerisation-based preparation platform, enabling synthesis of NPs with very similar size, size distribution, zeta potential and CBZ drug fraction – independent on monomer used. All batches were synthesised based on the same protocol, originally developed for synthesis of reference NPs. The only exception was NPs made from the 3-PCA monomer, which showed increased particle size and broadened size distribution compared to the other NPs. The 3-PCA NPs were therefore not chosen for further studies in this work, as the focus was to screen several new ACA materials to show their potential as building blocks for NPs for drug delivery, rather than optimising synthesis protocols specifically for all ACAs. 3-PCA could however, given its promising drug compatibility profile, be worth revisiting and optimisation, as it could allow for encapsulation of a wider array of APIs.

Encapsulation of the model substances CBZ and NR668 shows that encapsulation does not affect the physicochemical properties of the NPs. All monomers allowed for high CBZ drug fractions (>75%), indicating that only small amounts of drug are lost during synthesis – an important aspect when encapsulating expensive drugs. Additionally, it shows the NP capacity to encapsulate drug concentrations at levels relevant for preclinical studies (≥5%) and emphasises the PACA NP's potential as nanomedicines.

## 5 Conclusion

In the current work, the hypothesis was that the alcohols released as a degradation product from PACA polymers could be screened for cellular toxicity and used to select targets for previously untested ACA/PACAs. This could lead to an expansion of the materials suitable for use in PACA NPs with nanomedical applications. The process led to discovery of PEHCA NPs, prepared from 2-EHCA monomers using a miniemulsion polymerisation process. PEHCA NPs were found to be a promising candidate – showing an *in vivo* toxicity profile comparable to that of the go-to commercially alternative PEBCA. Additional studies involving drug compatibility revealed that 2-EHCA, 3-PCA and 2-PECA monomers can allow encapsulation of new compounds formerly regarded as incompatible with ACA materials in a miniemulsion polymerisation process. Encapsulation of the API cabazitaxel and the fluorescent dye NR668 was achieved in the new ACA/PACA systems for the first time. In summary, this work shows the value of expanding the ACA/PACA toolbox. Firstly, by identifying materials with reduced toxicity giving room for PACA NPs to expand beyond their primary use as anticancer therapies, to areas which require significantly more drug (and hence particle) to be delivered. Then secondly, by expanding the PACA applicability into new fields by broadening the range of compounds that can be readily encapsulated using a miniemulsion polymerisation process. Finally, it is anticipated that this work has provided the groundwork for these novel materials to be investigated for use in the wider PACA encapsulation methodology suite.

## CRediT authorship contribution statement

**Astrid Hyldbakk:** Writing – original draft, Methodology, Investigation, Formal analysis, Visualization. **Yrr Mørch:** Funding acquisition, Project administration, Methodology, Writing – review & editing. **Sofie Snipstad:** Funding acquisition, Project administration, Methodology, Investigation, Writing – review & editing. **Andreas K.O. Åslund:** Funding acquisition, Project administration, Methodology, Investigation, Writing – review & editing. **Geir Klinkenberg:** Conceptualization, Investigation. **Vu To Nakstad:** Investigation, Formal analysis, Writing – review & editing. **Ane-Marit Wågbø:** Investigation. **Ruth Schmid:** Conceptualization, Funding acquisition, Writing – review & editing. **Peter P. Molesworth:** Conceptualization, Funding acquisition, Investigation, Project administration, Methodology, Writing – review & editing.

## Declaration of Competing Interest

The authors declare the following financial interests/personal relationships which may be considered as potential competing interests:

Peter P. Molesworth reports financial support was provided by Research Council of Norway. Yrr Morch reports financial support was provided by Research Council of Norway. Sofie Snipstad reports financial support was provided by Research Council of Norway. Peter P. Molesworth has patent #Nanoparticles comprising copolymeric or homopolymeric compounds which comprise cyanoacrylate subunits (WO2020207655) pending to SINTEF TTO AS. Ruth Schmid has patent #Nanoparticles comprising copolymeric or homopolymeric compounds which comprise cyanoacrylate subunits (WO2020207655) pending to SINTEF TTO AS. Yrr Morch has patent #Nanoparticles comprising copolymeric or homopolymeric compounds which comprise cyanoacrylate subunits (WO2020207655) pending to SINTEF TTO AS.

## Footnotes

1 In this paper, the 4-MPCA term will be used due to confusion in the literature whether IHCA is short for isohexyl cyanoacrylate or 2-ethyl-butyl cyanoacrylate.
