# Supplementary material for: Identification of novel cyanoacrylate monomers for use in nanoparticle drug delivery systems prepared by miniemulsion polymerisation – A multistep screening approach
Source: Int J Pharm X. 2022 Jul 20;4:100124. doi: 10.1016/j.ijpx.2022.100124 (PMC9310130; doi:10.1016/j.ijpx.2022.100124)
Supplement: Supplementary file 1 — The following is the Supplementary material provided with this article. [file mmc1.docx]

# Appendix A. Supplementary data

**Table S1**: Alcohols, cyanoacrylates, chemical structure and abbreviations.

|  | *Alcohol*  *name* | *Alcohol* | *Monomer* | *Cyanoacrylate* |
| --- | --- | --- | --- | --- |
|  | 2-ethylhexanol |  | 2-EHCA |  |
|  | 1-heptanol |  | 1-HPCA |  |
|  | 3-methylbutanol |  | 3-MBCA | 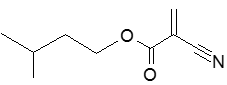 |
|  | 3-heptanol |  | 3-HPCA | 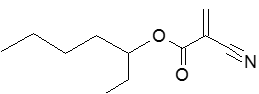 |
|  | Neopentanol | 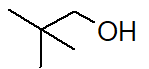 | NPCA | 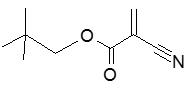 |
|  | 2-phenylethanol | 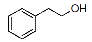 | 2-PECA |  |
|  | 1-pentanol |  | 1-PCA | 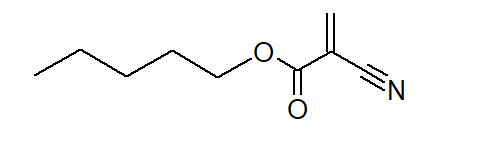 |
|  | 3-pentanol | 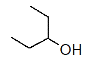 | 3-PCA |  |
|  | 3,3-dimethylbutanol | 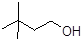 | 3,3-DMBCA | 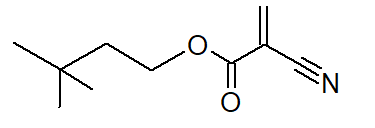 |
|  | 2-heptanol | 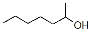 | 2-HPCA | 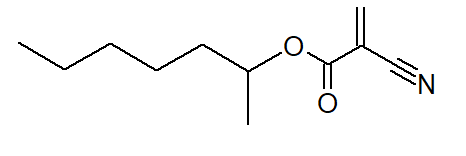 |
| Ctrl | 1-octanol |  | 1-OCA |  |
| Ctrl | 2-ethylbutanol | 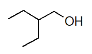 | 2-EBCA |  |
| Ctrl | 1-butanol | 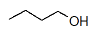 | 1-BCA | 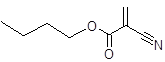 |
| Ctr | 4-methyl-pentanol | 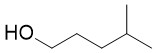 | 4-MPCA | 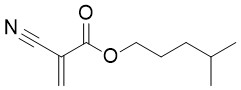 |

**Table S2**: Haematological parameters analysed in blood from rats injected with PEBCA and PEHCA NPs.

|  | | Control | PEBCA | | | | PEHCA | | | |
| --- | --- | --- | --- | --- | --- | --- | --- | --- | --- | --- |
|  |  |  | 4 hours | 3 days | 10 days | 16 days | 4 hours | 3 days | 10 days | 16 days |
| Number of animals | | 6 | 4 | 4 | 4 | 4 | 4 | 4 | 4 | 4 |
|  | | | | | | | | | | |
| *White Blood Cells* | | | | | | | | | | |
| Total count | *10^9^/L | 7.75±2.45 | 9.65±0.69 | 8.58±1.78 | 10.35±2.20 | 6.00±2.42 | 9.33±2.45 | 10.13±0.08 | 8.10±1.71 | 5.80±0.41 |
| Neutrophils | *10^9^/L | 0.68±0.25 | 3.55±0.48 | 1.73±0.67 | 1.43±0.22 | 0.75±0.19 | 4.35±1.65 | 2.10±0.61 | 1.43±0.52 | 0.85±0.19 |
| Lymphocytes | *10^9^/L | 6.68±2.41 | 5.45±0.60 | 6.40±1.28 | 8.25±2.09 | 4.95±2.36 | 4.53±0.91 | 7.33±0.74 | 6.15±1.36 | 4.68±0.59 |
| Monocytes | *10^9^/L | 0.15±0.05 | 0.43±0.05 | 0.18±0.05 | 0.28±0.13 | 0.08±0.05 | 0.35±0.13 | 0.27±0.06 | 0.23±0.05 | 0.08±0.05 |
| Eosinophils | *10^9^/L | 0.10±0.06 | 0.08±0.05 | 0.18±0.05 | 0.23±0.13 | 0.08±0.05 | 0.03±0.05 | 0.13±0.06 | 0.15±0.06 | 0.10±0.08 |
| Basophils | *10^9^/L | 0.017±0.041 | 0 | 0.025±0.050 | 0.075±0.050 | 0 | 0.025±0.050 | 0.033±0.058 | 0 | 0 |
|  |  |  |  |  |  |  |  |  |  |  |
| *Clotting potential* | | | | | | | | | | |
| Platelets | *10^9^/L | 1069.0±705.1 | 645.8±70.5 | 562.8±88.4 | 246.3±29.7 | 918.8±151.6 | 563.8±156.6 | 731.7±143.5 | 346.5±157.4 | 697.0±122.0 |
|  |  |  |  |  |  |  |  |  |  |  |
| *Red Blood Cells* | | | | | | | | | | |
| Total count | *10^12^/L | 8.23±0.66 | 9.30±0.91 | 8.30±0.50 | 11.80±0.32 | 7.28±0.86 | 10.20±0.84 | 8.10±0.10 | 10.55±0.52 | 7.93±1.26 |
| Haemoglobin (Hb) | g/L | 146.00±8.90 | 160.50±10.08 | 144.8±9.54 | 195.80±6.99 | 129.50±16.11 | 175.30±13.33 | 142.00±2.65 | 175.30±11.00 | 126.80±18.96 |
| Haematocrit | L/L | 0.46±0.05 | 0.53±0.04 | 0.46±0.03 | 0.67±0.02 | 0.41±0.06 | 0.57±0.05 | 0.45±0.01 | 0.59±0.04 | 0.43±0.07 |
| Mean Corp. Volume | fL | 56.17±2.56 | 56.25±0.96 | 55.25±0.96 | 56.50±1.73 | 55.50±1.73 | 56.00±0 | 56.00±1.00 | 56.00±2.16 | 54.50±2.08 |
| Mean Corp. Hb | g/L | 315.70±23.24 | 307.50±4.93 | 314.80±8.06 | 294.30±2.22 | 321.00±6.68 | 305.30±4.92 | 313.00±2.65 | 297.30±9.57 | 316.00±9.13 |
| Red Blood Cell Distribution Width | % | 12.83±0.98 | 13.25±0.50 | 13.50±0.58 | 14.00±0 | 13.50±0.58 | 12.50±0.57 | 14.00±0 | 14.25±0.50 | 13.00±0.82 |
|  |  |  |  |  |  |  |  |  |  |  |
| *Colour indicates statistical difference from saline control:* | | | | | |  | P ≤ 0.05 | P ≤ 0.01 | P ≤ 0.001 | P ≤ 0.0001 |

**Table S3**: Clinical chemistry parameters analysed in blood from rats injected with PEBCA and PEHCA NPs.

|  | | Control | PEBCA | | | | PEHCA | | | |
| --- | --- | --- | --- | --- | --- | --- | --- | --- | --- | --- |
|  |  |  | 4 hours | 3 days | 10 days | 16 days | 4 hours | 3 days | 10 days | 16 days |
| Number of animals | | 8 | 4 | 4 | 4 | 4 | 4 | 4 | 4 | 4 |
|  | | |  | | | |  | | | |
| *Electrolyte balance* | | |  | | | |  | | | |
| Sodium | mmol/L | 146.30±2.32 | 142.50±0.58 | 147.50±3.54 | 147.80±0.50 | 148.00±0 | 141.80±0.50 | 144.70±1.16 | 148.00±1.41 | 147.30±1.26 |
| Potassium | mmol/L | 4.04±0.28 | 4.40±0.22 | 4.05±0.21 | 4.05±0.31 | 3.78±0.13 | 4.15±0.25 | 4.27±0.12 | 3.98±0.26 | 4.00±0.28 |
| Calcium | mmol/L | 2.65±0.09 | 2.63±0.05 | 2.53±0.05 | 2.65±0.06 | 2.65±0.13 | 2.75±0.13 | 2.60±0 | 2.58±0.05 | 2.73±0.10 |
| Chloride | mmol/L | 108.90±2.36 | 104.30±0.96 | 109.50±3.54 | 111.00±1.16 | 111.30±1.26 | 103.80±1.26 | 106.30±1.16 | 111.80±1.26 | 111.30±0.96 |
|  |  |  |  |  |  |  |  |  |  |  |
| *Carbohydrate metabolism* | | |  | | | |  | | | |
| Glucose | mmol/L | 8.51±1.37 | 9.90±0.14 | 9.23±1.38 | 8.45±0.98 | 8.40±0.89 | 10.03±1.62 | 9.33±0.59 | 8.68±0.21 | 9.20±1.07 |
|  |  |  |  |  |  |  |  |  |  |  |
| *Pancreatic function* | | |  | | | |  | | | |
| Amylase | U/L | 2160.0±407.6 | 2081.0±320.1 | 1761.0±275.1 | 2247.0±231.9 | 1832.0±113.5 | 2203.0±196.1 | 1825.0±68.9 | 1938.0±244.5 | 1904.0±322.1 |
|  |  |  |  |  |  |  |  |  |  |  |
| *Liver function* | | |  | | | |  | | | |
| AST | U/L | 128.30±95.60 | 120.50±17.37 | 99.95±26.51 | 94.00±26.34 | 117.30±24.96 | 109.30±30.07 | 111.50±43.71 | 81.50±6.56 | 90.7±9.54 |
| ALT | U/L | 28.50±12.97 | 32.00±11.52 | 22.50±3.42 | 17.00±7.26 | 35.00±8.37 | 17.75±5.74 | 17.50±7.05 | 15.25±9.11 | 45.50±7.23 |
| AP | U/L | 138.9±29.49 | 115.50±31.33 | 128.00±11.17 | 111.30±21.05 | 135.00±23.17 | 86.25±27.78 | 159.80±39.14 | 114.00±31.78 | 161.00±31.38 |
| Total bilirubin | µmol/L | 0 | 0 | 0.25±0.50 | 0 | 0 | 0 | 0 | 0 | 0 |
|  |  |  |  |  |  |  |  |  |  |  |
| *Kidney function* | | |  | | | |  | | | |
| Creatinine | µmol/L | 38.00±4.34 | 41.25±3.10 | 30.50±2.38 | 35.00±3.46 | 37.75±0.50 | 35.25±4.72 | 39.25±3.69 | 32.00±2.16 | 35.50±2.08 |
| Urea | mmol/L | 6.09±0.79 | 7.83±0.62 | 5.38±1.23 | 4.68±0.30 | 5.83±0.67 | 5.05±0.53 | 5.85±0.96 | 4.95±0.17 | 5.90±0.79 |
|  |  |  |  |  |  |  |  |  |  |  |
| *Others* | | |  | | | |  | | | |
| Gamma-Glutamyl Transferase | U/L | 0 | 0 | 0 | 0 | 0 | 0 | 0 | 0 | 0 |
| Albumin | g/L | 30.00±2.21 | 27.13±1.09 | 22.00±0 | 27.60±1.65 | 29.17±0.68 | 31.33±1.24 | 24.08±0.82 | 26.55±1.20 | 29.97±1.82 |
| Total protein | g/L | 59.63±3.89 | 54.75±1.50 | 55.50±2.65 | 56.75±2.22 | 60.00±3.37 | 60.75±4.27 | 55.50±2.38 | 53.75±2.50 | 60.50±1.29 |
| Creatinine kinase | U/L | 658.4±604.8 | 541.8±156.0 | 293.0±158.7 | 333.5±167.7 | 381.8±113.7 | 668.0±145.8 | 444.5±332.4 | 224.8±60.0 | 307.0±147.9 |
| Inorganic phosphate | mmol/L | 1.86±0.33 | 2.20±0.08 | 1.98±0.05 | 2.13±0.10 | 1.70±0.25 | 2.48±0.22 | 2.05±0.10 | 2.00±0.14 | 1.48±0.21 |
|  |  |  |  |  |  |  |  |  |  |  |
| *Colour indicates statistical difference from saline control:* | | | | | |  | P ≤ 0.05 | P ≤ 0.01 | P ≤ 0.001 | P ≤ 0.0001 |


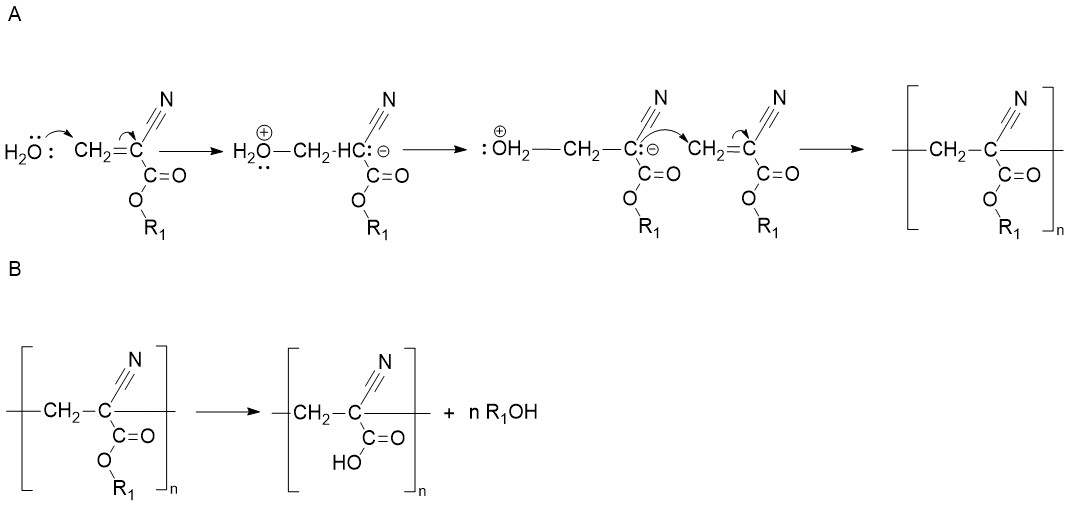


**Figure S1**: A schematic overview of (A) polymerisation and (B) degradation of PACA polymers.

**Figure S2**: Relationship between % viability after alcohol exposure and NP IC_50_ values in Hep G2 and LLC-PK1 cell lines by both LDH and MTT assays.

**Figure S3**: Organ-to-brain weight ratios.
